# Substantial Impact of COVID-19 on Self-Reported Mental Health of Healthcare Professionals in the Netherlands

**DOI:** 10.3389/fpubh.2021.796591

**Published:** 2022-01-10

**Authors:** Lars de Vroege, Anneloes van den Broek

**Affiliations:** ^1^Tranzo Department, Tilburg School of Social and Behavioral Sciences, Tilburg University, Tilburg, Netherlands; ^2^Department of Anxiety and Depression, GGz Breburg, Breda, Netherlands; ^3^Department of Post Academic Psychology Training and Education, Breburg Academy, GGz Breburg, Tilburg, Netherlands

**Keywords:** COVID-19, SARS-Cov-2, pandemic, mental health, healthcare professionals, the Netherlands

## Abstract

Initially, the COVID-19 pandemic caused a continued pressure on professionals working in hospitals due to the increase of affected patients. At the moment, the pandemic continues but thanks to all kinds of measures (e.g., social distancing) workload seems to decrease at the hospitals. On the contrary, patients with long-lasting symptoms due to COVID-19 infection or the pandemic begin to merge at the mental healthcare institutions in the Netherlands but this also holds true for other countries. Furthermore, healthcare professionals are affected by safety measures such as working from home, which led to an increased feeling of stress and may have led to a misbalance in work and private life. As a result, the question whether healthcare employees in mental healthcare experience impaired mental health remains unclear and chances are fair that mental health problems such as exhaustion and burnout may be prevalent. This study describes an online survey in which mental health amongst mental healthcare professionals is investigated. About 1,300 professionals from a large number of mental healthcare institutions replied the survey. Around 50% of the respondents experienced increased levels of stress. Feelings of anxiety, anger, and sadness were also increasingly experienced due to the COVID-19 pandemic. Furthermore, 4.2% replied that they were considering resigning their jobs which is alarming considering the shortage of healthcare professionals in mental healthcare institutions. The results support the importance of treatment or support of professionals in mental healthcare that experience psychological ailments.

## Introduction

Since 2019, the global COVID-19 pandemic is not dispensable in everyday life. Due to the widespread transmission, at the start of the pandemic a huge load of cases entered the hospitals and as a result the workload increased for healthcare professionals within the hospital. As the pandemic continued, more and more studies evaluated or started evaluating the impact of COVID-19 (1) on the mental health and well-being of these professionals. Heightened levels of stress, depression, and anxiety were already reported in the general population (2, 3). For professionals, the continuing pressure at work increases psychological pressure ultimately leading to feelings of helplessness, feelings of stress, irritability, and mental fatigue (4). Protection of the mental health of professionals working in healthcare has been identified as imperative (5) but has yet received little attention.

Several measures have been evoked by all affected countries such as lockdown to limit the spread of COVID-19 and the introduction of social distancing in public places which is considered standard procedure in case of a pandemic (6). Due to the nature of their work, professionals in healthcare are more exposed to infection, which means that such safety measures less apply for them. This may contribute to feelings of anxiety or other mental health symptoms. Furthermore, long hours and increased working load can contribute to feelings of exhaustion and tiredness which can ultimately contribute to the development of burn-out. Hence, the health of healthcare professionals during the pandemic is stressed by several authors (7–9). However, not only healthcare professionals but employees in healthcare in general may suffer from decreased mental health since the safety measures also apply on them. Furthermore, as the measurements decrease their workload also increased since waiting lists increased during the pandemic and (part of the) work is left behind. Also negative emotions, feelings of being inadequately supported are factors that may contribute mental burden (10) and confirmed or suspected cases of COVID-19 in the surrounding of professionals may further contribute to fear of disease.

Studies focusing on other pandemics already showed that the effect on mental health regarding stress, anxiety, and depression is substantial (11–16). Moreover, burnout and secondary traumatization were primarily reported by healthcare professionals primarily and were associated with increased depression and anxiety (17). Some studies also showed the same results amongst healthcare professionals in the COVID-19 pandemic (18, 19) and the need for specific interventions to support mental health due the pandemic is stressed (20). Due to staff shortage, in general, and the risk of developing burn-out, the consequences for professionals in mental healthcare due to the pandemic are pivotal. For instance, the psychological impact on professionals has severe negative consequences for institutions regarding patient care and, more in general, the healthcare system (21). Furthermore, the experience of negative emotions was related to poor work performance and resignation (22). However, information regarding mental health among mental healthcare professionals specific have not yet been comprehensively explored and reported in the Netherlands. Such survey is necessary to provide more mental healthcare for professionals which stress the need to understand the mental health of professionals in mental healthcare.

To the best of our knowledge, this is the first large study amongst mental health status in mental healthcare professionals across the Netherlands. This study examines mental health status regarding symptoms of anxiety, depression, anger, and sadness and levels of stress during the COVID-19 pandemic and explored the contribution of the pandemic to these feelings. Furthermore, we explored work status and plans to re-organize work (e.g., working less hours, considering quitting their job) due to the COVID-19 pandemic. Mental health support for professionals is much needed based upon previous investigations (8, 23). Such support of professionals in mental healthcare may be advised based upon the findings of this survey.

## Materials and Methods

### Study Design and Sample

We distributed an internet survey through social media (using LinkedIn) and send the survey to the board of directors of the (large) mental health institutions (with an average of 2,500 employees) of the Netherlands, Colleagues were asked to respond between august 4^th^ and September 11^th^ 2021. They survey was distributed using Qualtrics. We did not specifically focus on a specific type of employee within the mental health institutions so all employees that responded to our survey are referred to as professional within this article.

Initially, 1,399 responses were registered. 16 (1.1%) respondents did not start with the survey and 11 (0.9%) did not complete more than 50% of the questions and were therefore excluded from the study sample. Eventually, the sample included 1,372 respondents who stated to work within a mental health institution of which 74 (5.4%) did not want to respond to the question in which institution they were employed (i.e., wanted to remain anonymous). Their responses regarding experienced mental health were used nevertheless.

### Survey Questions and Distribution

Questions regarding experienced mental health during the COVID-19 pandemic included questions regarding the experience of increased symptoms of anxiety, sadness, levels of stress, sadness, and anger. Furthermore, three questions regarding work/private life balance were asked in order to evaluate the ability of professionals to balance life working from home. Three other questions were asked related to sick leave, taken days off, and absenteeism during the pandemic. Finally, we asked the respondents whether they were considering re-organizing their work (e.g., working less hours, quitting their job). Hence this survey was also used to evaluate mental health per institution (some of the institutions participate in a conjoint alliance), and due to the extent of the questions we decided to record the responses anonymously and therefore did not ask for age, gender, and other sociodemographic variables.

### Statistical Analyses

Categorical variables were presented by means of frequency tables. Comparisons between continuous variables were explored using paired-sample *t*-tests. We explored distribution differences between categorical variables with *Cramer's V*. The dataset was analyzed using the Statistical Package for the Social Sciences (SPSS) version 27.

## Results

### Experienced Mental Health Symptoms During COVID-19

Table 1 includes the results of the mental health symptoms exploration and the results of questions regarding balance between work and private.

**Table 1:** Mental health symptoms and balance work and private during the COVID-19 pandemic.

**Questions**						
**Compared to before the COVID-19 pandemic, i experience**						
	**More symptoms of** ***n*** **(%)**		**Not more/not less symptoms of** ***n*** **(%)**		**Less symptoms of** ***n*** **(%)**	**Missing** ***n*** **(%)**
Anxiety	262 (19.4)		1055 (78.3)		31 (2.3)	24 (1.8)
Depression	409 (30.3)		911 (67.5)		29 (2.1)	23 (1.7)
Higher levels of stress	658 (48.5)		650 (47.9)		49 (3.6)	15 (1.1)
Sadness	190 (14.2)		1119 (83.4)		33 (2.5)	30 (2.2)
Anger	318 (23.6)		994 (73.8)		35 (2.6)	25 (1.8)
**Compared to before the COVID-19 pandemic:**						
	Due to working from home, work and private balance has tipped the scale toward work					
	**Agree** ***n*** **(%)**	**Partly agree** ***n*** **(%)**	**Neutral** ***n*** **(%)**	**Partly disagree** ***n*** **(%)**	**Disagree** ***n*** **(%)**	**Missing** ***n*** **(%)**
	127 (9.4)	331 (24.6)	387 (28.7)	157 (11.7)	345 (25.6)	
						25 (1.8)
	I can less effectively balance work and private					
	**Agree** ***n*** **(%)**	**Partly agree** ***n*** **(%)**	**Neutral** ***n*** **(%)**	**Partly disagree** ***n*** **(%)**	**Disagree** ***n*** **(%)**	
	213 (15.7)	457 (33.7)	264 (19.5)	126 (9.3)	295 (21.8)	
						25 (1.8)
	I cannot balance (anymore) between work and private					
	**Agree** ***n*** **(%)**	**Partly agree** ***n*** **(%)**	**Neutral** ***n*** **(%)**	**Partly disagree** ***n*** **(%)**	**Disagree** ***n*** **(%)**	
	49 (3.6)	224 (16.7)	403 (30.0)	213 (15.8)	455 (33.9)	
						17 (1.2)

*missing values are displayed in last column and reflect missed responses on the according question and are calculated using the total sample of respondents (N = 1372)*.

First, most respondents (48.5%, *n* = 658) stated to experience higher levels of stress due to the pandemic and 30.3 % (*n* = 409) experienced more feelings of depression. More symptoms of anger was mentioned by 23.6% (*n* =318) respondents, 19.4% (*n* =262) respondents stated of experience more anxiety and 14.2% (*n* = 190) more sadness. Between 47.9 and 83.4% of the respondents stated that they did not experience either more or less symptoms of anxiety, depression, sadness, or anger or stress levels due to the pandemic. Less mental symptoms due to the pandemic were experienced by 2.1 to 3.6% of the respondents.

Respondents stated that they felt worse during the COVID-19 pandemic compared to before the pandemic on a 0–100 scale. In general, differences regarding self-reported level of mental health before the pandemic (*M* = 79.70, *SD* = 11.39) compared to during the pandemic (*M* = 70.96, *SD* = 14.62) were significantly worsened [*t*_(1360)_ = 22.06, *p* < 0.001, *Cohen's d* = 0.60]. More specifically, respondents stated that the COVID-19 pandemic significantly contributed to decreased mental health regarding anxiety (*Cramer's V* = *0.1*4*, p* = *0.0*04), depression (*Cramer's V* = *0.2*6*, p*< *0.0*01), higher levels of stress (*Cramer's V* = *0.3*0*, p*< *0.0*01), but not for sadness (*Cramer's V* = *0.1*6*, p*< *0.0*01) or anger (*Cramer's V* = *0.1*9*, p*< *0.0*01).

### Sick Leave and Absenteeism During COVID-19

Table 2 describes the results of questions regarding sick leave and absenteeism during the COVID-19 pandemic.

**Table 2:** Work-related questions regarding absenteeism and sick leave.

**Question**	**Which statement holds true regarding sick leave and/or absenteeism during the COVID-19 pandemic?**	
	I have taken more sick leave *n* (%)	Missing values *n* (%)
Agree	154 (11.3)	
Partly agree	122 (9.0)	
Neutral	109 (8.0)	
Partly disagree	47 (3.5)	
Disagree	925 (68.2)	
		15(1.1)
	I have taken more days off *n* (%)	
Agree	101 (7.5)	
Partly agree	168 (12.4)	
Neutral	137 (10.1)	
Partly disagree	70 (5.2)	
Disagree	875 (64.8)	
		21(1.5)
	I was more absent n (%)	
Agree	120 (8.8)	
Partly agree	106 (7.8)	
Neutral	119 (8.8)	
Partly disagree	57 (4.2)	
Disagree	954 (70.3)	
		14 (1.0)

*missing values are displayed in last column and reflect missed responses on the according question and are calculated using the total sample of respondents (N = 1372)*.

Between 68.2 and 70.3% stated that they did not taken more sick leave, more days off or were more absent during the COVID-19 pandemic. However, 11.3% (*n* = 154; agree) and 9.0% (*n* = 122; partly agree) reported taken more sick leave and 7.5% (*n* = 101; agree) and 12.4% (*n* = 168; partly agree) reported to have taken more days off. Between 7.8% (*n* = 106, partly agree) and 8.8% (*n* = 120; agree) stated to be more absent during the pandemic.

28.6% (*n* = 298) and 27.8% (*n* = 290) of the respondents stated that the COVID-19 pandemic contributed to or contributed to some extent, respectively, to their experienced (decreased) mental health during the participation in this survey. 23.7% (*n* = 247) stated that the COVID-19 pandemic contributed a little to their well-being and 19.9% (*n* =208) stated that it did not.

### Work-Related Results

Table 3 describes the results regarding intentions to organize work differently. 101 respondents stated to consider working less hours (7.5%) and 56 (4.2%) stated considering quit working in healthcare. Four hundred and twenty-fifth respondents (31.5%) stated to consider working more from home whilst 767 (56.9%) replied that they did not consider reorganizing their working hours and/or situation.

**Table 3:** Reorganization of work.

**Question**	**Due to the COVID-19 pandemic, I consider to reorganize my work** ***n* (%)**	**Missing values** ***n* (%)**
No, I don't consider reorganizing my work	767 (56.9)	
Yes, I consider working more from home	425 (31.5)	
Yes, I consider quitting working in health care	56 (4.2)	
Yes, I consider working less hours	101 (7.5)	
		23 (1.7)

*missing values are displayed in last column and reflect missed responses on the according question and are calculated using the total sample of respondents (N = 1372)*.

## Discussion

This national-based online survey reported the prevalence of mental health status of employees in mental healthcare in the Netherlands. Our study shows that employees in mental healthcare reported high levels of stress (about 50%), and more symptoms of depression (30%), and anxiety (14%). Based on the finding, these symptoms were primarily the result of the COVID-19 pandemic. In general, respondents also stated they were considering re-organizing their work by either working less hours (7.5%) or working more from home (31.5%). 4.2% were considering resignation. Findings from the current study stress the need for professional support of professionals in mental healthcare. The precariousness of the healthcare system warrants governmental and organizational policies during the continuation of the COVID-19 pandemic.

A recent large systematic review in the Lancet (24) showed that due to the COVID-19 pandemic, prevalence rates of (major) depressive disorder and anxiety disorder increased substantially. Furthermore, their results showed that females were more affected by the pandemic than males with regards to depressive and anxiety disorder. Since more females than males are working in mental health, these results are alarming with regard to sick leave and/or unemployment due to mental illness. Furthermore, our results are in line with the results of other studies (11, 13, 14) but also compared to the results of previous pandemics (12) which further stresses the need providing aid to healthcare workers.

This is the first study that explored mental health status of Dutch professionals in mental healthcare with a large response rate. This survey ensured maximum anonymity of the respondents. Prior to distributing the survey, we accepted this limitation in light of our opinion that it would increase responses. Furthermore, small numbers of missing values can be reported. 1–2.2% of the initial respondents did not complete or did not answer the specific question. One other limitation of this study concerns its cross-sectional characteristic, conclusions regarding causal contributions of COVID-19 pandemic or change over time of mental health status during the pandemic cannot be made. Furthermore, because of the accepted anonymity of our study, we are unable to follow-up individual responses throughout the rest of the pandemic which limited us in tracking individual changes over time. However, a future survey will be conducted to further explore long-term effects of the pandemic regarding mental health status since we know from previous pandemics that the development of posttraumatic stress disorder and/or burn-out can be prevalent (25).

In general, working in healthcare during pandemic times can be distressful for instance because of participation in acts that may transgress personal values, beliefs, and/or morals and has gained attention during COVID-19 (26, 27). Healthcare employees are sometimes faced with dilemmas without a “right” solution. Employees working in healthcare experience (moral) distress which they related to conflicts between what they believed was right during the COVID-19 pandemic and institutional constraints (28, 29). This result shows the need for institutional meddling to prevent emotions such as overwhelm, fear, frustration and feelings of hopelessness, and helplessness. Since these emotions and feelings are associated with significant mental health challenges such as mental illnesses, behavioral issues (e.g., substance misuse), physical health challenges (e.g., sleep disturbances), and occupational impairment (e.g., burn-out and work absenteeism) (30).

Strategies for combating COVID-19 were primarily focused on the general health of the population, whilst the consequences of the pandemic become clearer and continue to develop strategies should yet also incorporate prevention and treatment of professionals in mental healthcare. Both government and healthcare institutions have the responsibility to protect their professionals regarding mental health. Prior studies from other pandemics show that the context of the institution has strong effects on psychological outcomes for the workforce (31). Furthermore, psychological support, focused on organizational but also individual characteristics is necessary (25). Social support outside the work environment, maintenance of social contact, but also clear communication and precautionary measures reduce the likelihood of emotional distress (31) and ultimately work absenteeism. Furthermore, promotion of self-care is much needed. Moreover, a study reported that professionals in healthcare have requested five things from managers during the pandemic: “hear me, protect me, prepare me, support me, and care for me” (32). Providing mental healthcare to their co-workers can be part of the psychosocial support that will create or increase resilience. Preparing young professionals during training how to cope with stressful experiences will prevent them from developing burn-out. Prior, or during, the development of such support, one should address stigma of being treated by a colleague thus creation of a safe environment, requires clear organizational strategies for mental health status of the staff, consistent and clear communication.

## Conclusion

This study shows a high level of distress, anxiety, and depression amongst employees in mental healthcare in the Netherlands. The high level of distress (almost 50%) is worrying because of the possible development into burn-out. The fact that these high levels of distress may ultimately lead to sick leave or unemployment due to mental illness is also worrying. Furthermore, the heightened levels of anxiety and depression further warrant mental support to prevent work absenteeism due to, for instance, post-traumatic stress disorder or burn-out. Promotion of self-care is needed, as is psychosocial support which both require organizational support of staff and clear consistent communication to provide a good working environment. Due to the fact that the pandemic did not reach its end yet, it is expected that the pressure on healthcare will continue within the coming decades. Therefore, it is important to pay attention to self-care, work-life balance, and job satisfaction in the training of healthcare professionals.

## Data Availability Statement

The raw data supporting the conclusions of this article will be made available by the authors upon request.

## Ethics Statement

The participants of this study provided consent obtained through the internet. The study protocol was approved by the scientific review committee of GGz Breburg (file number CWO 2021-35).

## Author Contributions

All authors listed have made a substantial, direct, and intellectual contribution to the work and approved it for publication.

## Conflict of Interest

The authors declare that the research was conducted in the absence of any commercial or financial relationships that could be construed as a potential conflict of interest.

## Publisher's Note

All claims expressed in this article are solely those of the authors and do not necessarily represent those of their affiliated organizations, or those of the publisher, the editors and the reviewers. Any product that may be evaluated in this article, or claim that may be made by its manufacturer, is not guaranteed or endorsed by the publisher.

## References

[1] Nochaiwong S Ruengorn C. Mental health circumstances among health care workers and general public under the pandemic situation of COVID-19 (HOME-COVID-19). Medicine. (2020) 99:e20751. 10.1097/MD.0000000000020751 32590751 PMC7329008

[2] Wang C Pan R Wan X Tan Y Xu L Ho CS. Immediate Psychological Responses and Associated Factors during the Initial Stage of the 2019 Coronavirus Disease (COVID-19) Epidemic among the General Population in China. Int J Res Public Health. (2020) 17:1729. 10.3390/ijerph17051729 32155789 PMC7084952

[3] Zhu Y Chen L Ji H Xi M Fang Y Li Y. The risk and prevention of novel coronavirus pneumonia infections among inpatients in psychiatric hospitals. Neurosci Bull. (2020) 36:299–302. 10.1007/s12264-020-00476-9 32096116 PMC7056754

[4] Huang JZ Han MF Luo TD Ren AK Zhou XP. [Mental health survey of medical staff in a tertiary infectious disease hospital for COVID-19]. Chinese journal of industrial hygiene and occupational diseases. (2020) 38:192–5. 10.3760/cma.j.cn121094-20200219-00063 32131151

[5] World Health Organization. Mental Health and Psychosocial Considerations During the Covid-19 Outbreak. (2020). Available online at: https://www.who/int/docs/default-source/coronaviruse/mental-health-considerations.pdf (accessed October 8, 2021)

[6] Wilder-Smith A Freedman DO. Isolation, quarantine, social distancing and community containment: pivotal role for old-style public health measures in the novel coronavirus (2019-nCoV) outbreak. J Travel Med. (2020) 27:taaa020. 10.1093/jtm/taaa020 32052841 PMC7107565

[7] de Vroege L Gribling G van den Broek A. ['Don't forget yourself when taking care of others' - mental support for health care professionals during the COVID-19 crisis]. Tijdschrift voor psychiatrie. (2020) 62:424–6. Available online at: https://www.tijdschriftvoorpsychiatrie.nl/assets/articles/62-2020-6-artikel-devroege.pdf 32902835

[8] de Vroege L van den Broek A. Results of mental support for health care professionals and mental care during the COVID-19 pandemic. J Public Health. (2021) 43:490–2. 10.1093/pubmed/fdaa278 33501979 PMC7928758

[9] Sandesh R Shahid W Dev K Mandhan N Shankar P Shaikh A. Impact of COVID-19 on the Mental Health of Healthcare Professionals in Pakistan. Cureus. (2020) 12:e8974. 10.7759/cureus.8974 32775056 PMC7402435

[10] Chen Q Liang M Li Y Guo J Fei D Wang L. Mental health care for medical staff in China during the COVID-19 outbreak. Lancet Psychiatry. (2020) 7:e15–6. 10.1016/S2215-0366(20)30078-X 32085839 PMC7129426

[11] Muller AE Hafstad EV Himmels JPW Smedslund G Flottorp S Stensland S. The mental health impact of the covid-19 pandemic on healthcare workers, and interventions to help them: A rapid systematic review. Psychiatry Res. (2020) 293:113441. 10.1016/j.psychres.2020.113441 32898840 PMC7462563

[12] Lee SM Kang WS Cho AR Kim T Park JK. Psychological impact of the 2015 MERS outbreak on hospital workers and quarantined hemodialysis patients. Compr Psychiatry. (2018) 87:123–7. 10.1016/j.comppsych.2018.10.003 30343247 PMC7094631

[13] Suryavanshi N Kadam A. Mental health and quality of life among healthcare professionals during the COVID-19 pandemic in India. Brain Behav. (2020) 10:e01837. 10.1002/brb3.1837 32918403 PMC7667343

[14] Rahman MA Rahman S Wazib A Arafat SMY Chowdhury ZZ Uddin BMM. COVID-19 Related Psychological Distress, Fear and Coping: Identification of High-Risk Groups in Bangladesh. Front Psychiatry. (2021) 12:718654. 10.3389/fpsyt.2021.718654 34484005 PMC8414638

[15] Xiong J Lipsitz O Nasri F Lui LMW Gill H Phan L. Impact of COVID-19 pandemic on mental health in the general population: A systematic review. J Affect Disord. (2020) 277:55–64. 10.1016/j.jad.2020.08.001 32799105 PMC7413844

[16] Wang Y Kala MP Jafar TH. Factors associated with psychological distress during the coronavirus disease 2019 (COVID-19) pandemic on the predominantly general population: A systematic review and meta-analysis. PLoS ONE. (2020) 15:e0244630. 10.1371/journal.pone.0244630 33370404 PMC7769562

[17] Buselli R Corsi M. Professional Quality of Life and Mental Health Outcomes among Health Care Workers Exposed to Sars-Cov-2 (Covid-19). Int J Environ Res Public Health. (2020) 17:6180. 10.3390/ijerph17176180 32858810 PMC7504107

[18] Li Z Ge J Yang M Feng J Qiao M Jiang R. Vicarious traumatization in the general public, members, and non-members of medical teams aiding in COVID-19 control. Brain Beh Immun. (2020) 88:916–9. 10.1016/j.bbi.2020.03.007 32169498 PMC7102670

[19] Ornell F Schuch JB. “Pandemic fear” and COVID-19: mental health burden and strategies. Braz J Psychiatry. (2020) 42:232–5. 10.1590/1516-4446-2020-0008 32267343 PMC7236170

[20] Rahman MA Hoque N Alif SM. Factors associated with psychological distress, fear and coping strategies during the COVID-19 pandemic in Australia. Global Health. (2020) 16:95. 10.1186/s12992-020-00624-w 33032629 PMC7542573

[21] Patel RS Bachu R Adikey A Malik M Shah M. Factors related to physician burnout and its consequences: a review. Behav Sci. (2018) 8:98. 10.3390/bs8110098 30366419 PMC6262585

[22] Bai Y Lin CC Lin CY Chen JY Chue CM Chou P. Survey of stress reactions among health care workers involved with the SARS outbreak. Psychiatr Serv. (2004) 55:1055–7. 10.1176/appi.ps.55.9.1055 15345768

[23] De Vroege L Bruins D Van den Broek A. [Health care professionals, don't forget yourself!]. GZ-psychologie. (2021) 13: 20–4. 10.1007/s41480-021-0823-232902835

[24] Collaborators C-MD. Global prevalence and burden of depressive and anxiety disorders in 204 countries and territories in 2020 due to the COVID-19 pandemic. Lancet. (2021) 398:1700–12. 10.1016/S0140-6736(21)02143-7 34634250 PMC8500697

[25] Maunder RG Leszcz M Savage D Adam MA Peladeau N Romano D. Applying the lessons of SARS to pandemic influenza: an evidence-based approach to mitigating the stress experienced by healthcare workers. Can J Public Health. (2008) 99:486–8. 10.1007/BF03403782 19149392 PMC5148615

[26] Williamson V Murphy D Greenberg N. COVID-19 and experiences of moral injury in front-line key workers. Occup Med. (2020) 70:317–9. 10.1093/occmed/kqaa052 32239155 PMC7184422

[27] Rushton CH Thomas TA Antonsdottir IM Nelson KE. Moral Injury and Moral Resilience in Health Care Workers during COVID-19 Pandemic. J Palliat Med. (2021) 22. 10.1089/jpm.2021.0076 PMC9081047 34678091

[28] Lake ET Narva AM Holland S Smith JG. Hospital nurses' moral distress and mental health during COVID-19. J Adv Nurs. (2021) 17. 10.1111/jan.15013 PMC8447301 34402538

[29] Cacchione PZ. Moral Distress in the Midst of the COVID-19 Pandemic. Clin Nurs Res. (2020) 29:215–6. 10.1177/1054773820920385 32363981

[30] Hall NA Everson AT Billingsley MR Miller MB. Moral injury, mental health and behavioural health outcomes: A systematic review of the literature. Clin Psychol Psychother. (2021). 10.1002/cpp.2607 33931926

[31] Chan AO Huak CY. Psychological impact of the 2003 severe acute respiratory syndrome outbreak on health care workers in a medium size regional general hospital in Singapore. Occup Med. (2004) 54:190–6. 10.1093/occmed/kqh027 15133143 PMC7107861

[32] Shanafelt T Ripp J Trockel M. Understanding and addressing sources of anxiety among health care professionals during the COVID-19 pandemic. JAMA. (2020) 323:2133–4. 10.1001/jama.2020.5893 32259193

